# The Diagnostic and Therapeutic Potential of Oligonucleotide Aptamers in Alzheimer’s Disease

**DOI:** 10.3390/cells14181424

**Published:** 2025-09-11

**Authors:** Georgios Katsipis, Eleni E. Tzekaki, Sophia Iasonidou, Anastasia A. Pantazaki

**Affiliations:** 1 Laboratory of Biochemistry, Department of Chemistry, Aristotle University of Thessaloniki, 54124 Thessaloniki, Greece; katsiphs.g1@gmail.com (G.K.); etzekaki@chem.auth.gr (E.E.T.); iasonidoussofia@gmail.com (S.I.); 2 Laboratory of Neurodegenerative Diseases (LND), Center for Interdisciplinary Research and Innovation, Aristotle University of Thessaloniki, 57001 Thermi, Greece

**Keywords:** aptamers, Alzheimer’s disease, diagnosis, biomarkers, nanotechnology

## Abstract

Alzheimer’s disease (AD) is the neurodegenerative condition with the outmost future challenges, with timely diagnosis and treatment being the most urgent. Discovery of more and more biomarkers is widely attempted; however, current diagnostic methods often lack sensitivity, specificity, and accessibility. Nucleotide aptamers—short, highly specific oligonucleotide or ligands—are now recognized as highly promising molecular agents for both measuring and targeting key AD biomarkers, with the most notorious being amyloid-beta (Aβ), tau protein, and disease-associated microRNAs (miRNAs). This review provides a comprehensive analysis of nucleotide aptamers related to AD, detailing their mechanisms of selection, recent advances in biosensing applications, and therapeutic potential. Aptamers, targeting the most significant biomarkers of AD, are mainly discussed, as well as ones interacting with novel, promising biomarkers, with a special aim on miRNAs. Additionally, aptamers are compared with conventional antibody-based approaches, highlighting their advantages in terms of stability, cost-effectiveness, and ease of modification. By elucidating the role of aptamers in AD diagnosis and treatment, this review underscores their promise as next-generation tools for precision medicine and neurodegenerative disease management.

## 1. Introduction

Dementia is regarded as the sixth cause of fatality worldwide (4.4%), with the U.S. recording a certified 121,499 Alzheimer’s disease (AD) deaths in 2019. AD is a progressive, generally irreversible neurodegenerative disorder and is the most common cause of dementia, estimated at about 60–80% of cases [1,2,3]. It is estimated that one in three elderly people over 85 worldwide suffer from some form of dementia, with AD primarily blamed for disability in people over 60, with a survival window of 3–6 years [4,5,6]. The value of statistical life (VSL) based societal economic burden of Alzheimer’s disease and related dementias was estimated at US$2.8 trillion in 2019, rising to US$16.9 trillion by 2050 (VSL approach), equivalent to roughly a six-fold increase [7]. Therefore, early diagnosis as well as follow-up clinical evaluation of patients is expected to offer multiple social and economic benefits [1,2,3].

The time required for full-blown AD symptoms is a factor of age, genetics, gender, educational level, etc. [8]. It is supposed that a long presymptomatic phase is usually followed by Mild Cognitive Impairment (MCI)—a condition lasting for 2–7 years, in which short-term memory problems appear due to neuronal loss in the hippocampus. For vaguely 40–75% of MCI patients, the pathology progresses to full-blown AD. In this last phase, there is an immense mental, physical, and financial burden on the patients and caregivers, and no significant amelioration to the patient’s condition can be implemented. Therefore, it is urgent to diagnose AD at the first or even pre-clinical stages [6,9,10].

The brain of individuals suffering with AD presents severe atrophy, while microscopically the pathological characteristics include aggregation of extracellular neuronal beta-amyloid protein (Aβ) plaques and intraneuronal neurofibrillary tangles (NFTs) of hyperphosphorylated tau protein (p-tau). The etiology for abnormal aggregation and the incomplete clearance of those is still unclear. However, it is generally acceptable that aggregation is accompanied by neurotoxic side effects such as extensive inflammation in the brain (neuroinflammation), oxidative stress, disruption of neurotransmission, of the blood-brain barrier (BBB) integrity, of the cerebral blood circulation, and degeneration of neuronal cells and axons [11,12].

As to date AD treatment has been inadequate or even with life-threatening side effects, early diagnosis is regarded as the key for preventing the progression of impending dementia and its subsequent effects. Next-generation materials have emerged as powerful tools for diagnostic and therapeutic purposes [13,14]. Among various agents, aptamers are acknowledged as outstanding candidates. Aptamers are oligonucleotide (RNA/DNA) or peptide molecules that are highly sensitive and selective against various targets, like proteins, nucleotides, or even antibiotics, small molecules, and cells [15]. Aptamers were first introduced in 1990 as sensitive and specific RNA ligands for the binding of organic dyes and the DNA polymerase of the T4 virus [16,17]. Three decades since these breakthroughs, oligonucleotide aptamers are now widely explored by researchers to overcome the diagnostic challenges of AD, and even its possible therapy [18,19,20]. Aptamers are regarded as very promising tools as they possess several advantages, i.e., specificity; small size, which gives them the ability to pass through the BBB [21,22,23]; thermal stability; easy chemical modification without affecting their abilities; and low-cost production with minimal ethical considerations [24].

**Figure 1:**
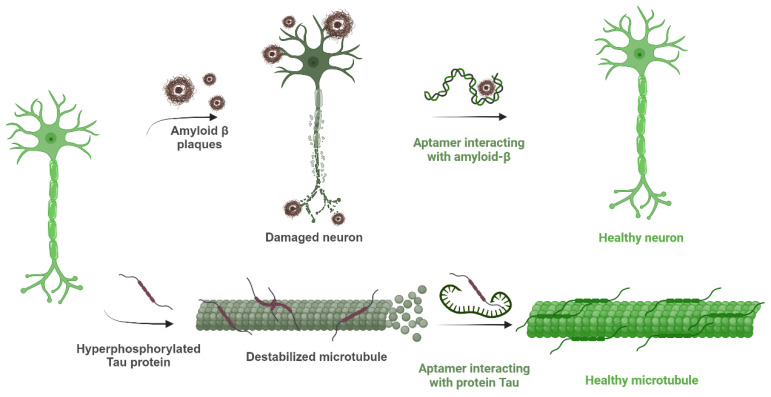
Schematic description of the consequences of amyloid-β plaques and hyperphosphorylated tau protein in Alzheimer’s disease and the importance of aptamers blocking the effects of those substances by interacting with them. Created with BioRender (BioRender.com, 2025).

In the last two decades, several oligonucleotide molecules were studied as potential aptamer targets for alleviating AD-associated neuropathology. The amyloid peptides Aβ_40_ and Aβ_42_ and the oligomeric form of Aβ peptides are probably at the epicenter of these studies, with both DNA and RNA aptamers described for detecting the peptides in biological fluids or inhibiting their aggregation [25,26,27,28]. Aptamers are also currently synthesized for targeting tau protein and several of its phosphorylated isoforms [18,29,30,31]. The possible disrupting effect of aptamers against the Aβ- or p-tau-induced neurotoxicity is depicted in Figure 1. Cutting-edge biosensors implicating the use of antibodies, aptamers, and nanotechnology for extra-sensitive analyses [18,27,32] and in vivo or ex vivo imaging [33,34] are some of the many applications of aptamer technology that are currently under development for the early and specific diagnosis of the disease. In the current review, the employability of oligonucleotide aptamers (DNA or RNA) in AD diagnosis and therapy is discussed. As peptide aptamers are a less common aptamer family, while also sharing some common disadvantages with the antibodies, they were not included in this presentation.

## 2. Key Biomarkers of Alzheimer’s Disease Pathology

Biomarkers serve as critical indicators of biological processes, as their measurement can determine the AD staging and provide rich data associated with possible amelioration due to experimental medications. Defined as molecular, biochemical, or imaging markers, they are the means of quantification, monitoring, and forecasting disease evolution, as well as in gauging treatment response. Biomarkers are increasingly integrated into clinical settings for diagnostic support, therapeutic decision-making, and disease activity assessment. Their significance extends into drug development, where they assist in patient stratification, monitoring treatment efficacy and safety, and optimizing dosing protocols [35].

### 2.1. Key AD Biomarkers in Biological Fluids

Aβ aggregation, tau protein hyperphosphorylation, and the neuronal accumulation of phosphorylated tau are possibly the most crucial molecular events associated with AD progression. These pathologies are primarily and predominantly manifested in the brain hippocampus. Cerebrospinal fluid (CSF) and plasma provide accessible means for investigating biochemical alterations in the central nervous system (CNS), given the impracticality of direct brain biopsies or microdialysis [36]. From extensive biochemical and immunohistological analyses of AD brain tissue, key proteins such as Aβ_40_, Aβ_42_, tau, and their phosphorylated variants (p-tau) are widely proposed as robust indicators for the diagnosis and prognosis of the disease [37,38].

**Figure 2:**
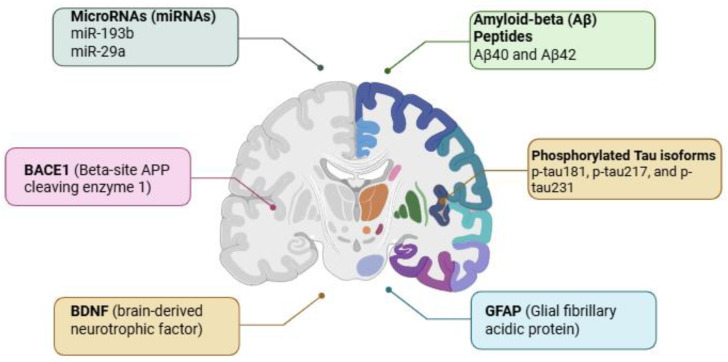
Illustration of Alzheimer’s disease biomarkers and their associated brain regions. Amyloid-beta (Aβ) peptides (Aβ_40_ and Aβ_42_) and the isoforms of the phosphorylated tau protein (p-tau181, p-tau217, and p-tau231) accumulate predominantly in areas related to cognitive function and memory. Concurrently, elevated levels of BACE1 (beta-site APP cleaving enzyme 1), GFAP (glial fibrillary acidic protein), BDNF (brain-derived neurotrophic factor), and dysregulated microRNAs (miR-193b, miR-29a) are observed. These molecular markers serve as critical indicators of neurodegeneration, inflammation, synaptic dysfunction, and overall disease progression. Their detection in cerebrospinal fluid and peripheral blood offers a non-invasive window into the pathological processes of AD, facilitating early diagnosis and therapeutic monitoring. Created with BioRender (BioRender.com, 2025).

Several other biomarkers are also recognized for their possible involvement in AD pathology and considered as promising for diagnostic or therapeutic targets (Figure 2). In the current review, we will focus on aptamers for Aβ peptides, tau protein, or p-tau isoforms, while a short presentation for the possible role of miRNAs is also included.

#### 2.1.1. Aβ Peptides as Biomarkers

The typical association of Aβ peptides with amyloid plaques is the amyloid with 40 residuals of amino acids and 42 residuals, with Aβ_42_ regarded as more cytotoxic due to its extensive tendency for aggregation. Aβ peptides are produced by successive proteolysis of a larger precursor peptide called the amyloid precursor protein (APP). Enzymes called secretases, mainly beta-secretase 1 (BACE1) and gamma-secretase (Presenilins, PSEN), are implicated in the so-called “amyloidogenic processing” of APP [11,12]. 

Brain Aβ species are cleared through all biological fluids, with most studies aiming at measuring their levels in the cerebrospinal fluid (CSF) and blood plasma. While Aβ_40_ is more abundant, Aβ_42_ has a central role to the “amyloid cascade hypothesis” and is more diagnostically valuable [39]. In AD individuals, Aβ_42_ is significantly diminished in CSF and plasma compared to age-matched controls, a reduction attributed to the peptide’s sequestration within amyloid plaques [40]. This decline in Aβ_42_ levels aligns with PET findings and postmortem brain Aβ assessments, indicating its direct relation to amyloid plaque burden [41]. In addition, the ratio of Aβ_42_/Aβ_40_ in blood plasma is now regarded as a very promising, easily measurable biomarker [42].

#### 2.1.2. Tau Proteins and Their Role in AD Pathophysiology

Tau proteins, especially p-tau, are equally important in AD diagnostics. Although the exact isoforms of tau in CSF and blood and their transport mechanisms are not fully elucidated, evidence suggests that neuronal damage and neurofibrillary tangles are the primary sources of their presence in the biological fluids [39,43]. Increased titers of tau peptides in the CSF are often present before clinical symptoms manifest. These biomarkers monitor the disease stage but also offer insights into disease progression [44,45]. 

Phosphorylation of tau protein is physiological for its role as a stabilizer of microtubules. However, the aberrant hyperphosphorylation is regarded as one of the main contributors to neurodegeneration, due to the formation of abnormal NFTs. There are several p-tau species studied as potential biomarkers in CSF and blood plasma, with the most prominent being p-tau181, p-tau217, and p-tau231 [46]. Especially, p-tau217 is regarded as the most promising blood p-tau species, as it seems to correlate significantly with tau-PET-positivity and can perform an accurate diagnosis of the disease in symptomatic individuals [47,48]. Recently, the U.S. Food and Drug Administration (FDA) has approved the ratio of p-tau217 against Aβ_42_ in blood plasma as a very sensitive biomarker for early diagnosis of AD [49].

## 3. Challenges in AD Diagnosis and Future Directions

The high costs for techniques like PET and MRI present significant barriers for the accurate detection of AD, leading to misdiagnosis. The hunt for tiny brain changes provides the cutting-edge of early diagnosis to elucidate clinical symptoms with a combination of neuroimaging methods, state-of-the-art biomarkers, and learning machinery to specify an appropriate treatment to impede the disease process [50]. Recent studies suggest that plasma biomarkers, such as p-tau217, could significantly reduce the need for costly PET scans by approximately 57%, making diagnosis more accessible and cost-effective [44].

In 2014, Kiddle et al. reviewed 163 potential blood biomarkers for AD but only 9 were consistently associated with AD phenotypes [51]. Although promising, none of these blood-based biomarkers have yet been validated for widespread clinical use. However, since 2018, validated biomarkers, including CSF concentrations of Aβ_42_, the Aβ_42_/Aβ_40_ ratio, p-tau/Aβ_42_, total tau, and p-tau181, have been introduced into clinical practice, offering greater diagnostic clarity [52].

## 4. Chemistry, Design, and Synthesis of Oligonucleotide Aptamers

### 4.1. Aptamer Function

Oligonucleotide aptamers are short nucleic acid sequences, composed of either DNA or RNA and typically range from 20 to 100 nucleotides in length, that exhibit high affinity and specificity toward distinct target molecules or classes of molecules. They display dissociation constants (Kd) that range from low pM to μM [53,54], may exhibit off-target interactions under certain conditions [55], and are often characterized as “chemical antibodies”. However, several limitations remain to be resolved—unmodified aptamers are susceptible to nucleases, can show off-target binding if not fully optimized, and often require delivery strategies to achieve durable in vivo activity [56]. For these reasons, current research combines biochemical selection, structural/biophysical characterization, and chemical modification to maximize specificity, stability, and translational potential.

**Figure 3:**
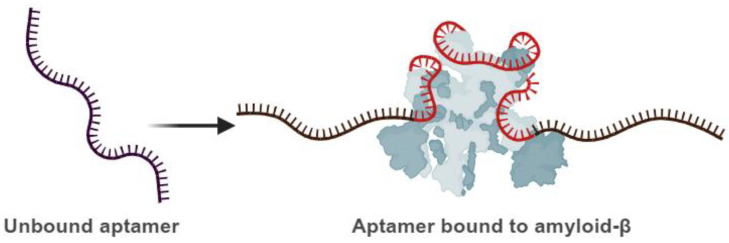
Schematic illustration of the aptamer’s capability to undergo conformational changes. **Left**: unbound aptamer. **Right**: bound aptamer to the target molecule (the protein molecule illustrated with grey color). An aptamer that conformationally changes and binds its target is in red, while the stable parts are in black. Created with BioRender (BioRender.com, 2025).

Aptamers have an innate quality to change their structure in secondary folds. As a result, they generate a three-dimensional (3D) model crucial in driving recognition-based binding potency between an aptamer sequence and its corresponding specific target. Hydrophobic and electrostatic interactions in conjunction with hydrogen bonds, van der Waals forces, shape complementarity, and stacking are some of the reactions that occur between aptamers and target molecules [57]. A crude representation of how the 3D-folding of aptamers drives the interaction with Aβ peptide is provided in Figure 3. A growing body of mechanistic work shows that aptamers act on Aβ and tau primarily by selective molecular recognition of pathogenic conformers and by physically blocking or redirecting aggregation pathways. For Aβ, high-affinity RNA aptamers selected against protofibrils (e.g., E22P-AbD43) form specific secondary/tertiary motifs (G-quadruplex/loop structures) that permit tight binding to monomer/dimer units and protofibrillar surfaces; binding of E22P-AbD43 suppresses nucleation and redirects aggregation to less-toxic spherical assemblies, with a corresponding reduction of neurotoxicity in cellular assays [58]. Computational studies (i.e., molecular docking) of aptamer–target complexes support these observations by identifying stable binding poses and interaction hotspots—typically hydrophobic patches plus basic residues on the protein—and by yielding binding free-energy estimates that correlate with experimental Kd values, providing a plausible structural basis for aptamer selectivity [59,60]. In addition, fluorescently labeled RNA aptamers (e.g., β55) demonstrate highly specific plaque/oligomer labeling in sectioned human tissue and in vivo AD animal models, confirming that aptamer binding is conformation-selective and can be exploited for targeted imaging and delivery [61]. For tau, selected aptamers have been shown to bind phosphorylatable epitopes or aggregation-prone tau species and to inhibit phosphorylation-dependent oligomerization in vitro, indicating a mechanism in which aptamer binding prevents inter-molecular tau–tau interfaces required for nucleation and fibril elongation [62].

RNA or DNA sequences are now widely studied as promising co-factors in the field of diagnosis, treatment, drug delivery, and imaging [19]. Aptamers are now employed as the recognizing element of electrical/electrochemical sensors (surface plasmon resonance [63], surface-enhanced Raman [64], optical [65], and colorimetric sensors [66]), or even as therapeutic, drug delivery, or imaging probes [57,67,68,69,70].

### 4.2. Aptamer Acquiring Through the Systematic Evolution of Ligands by Exponential Enrichment

**Figure 4:**
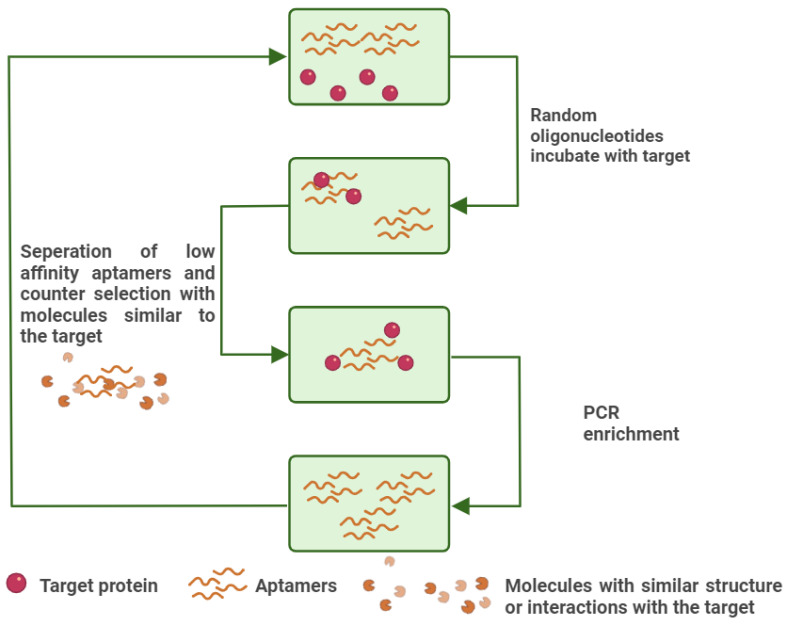
An illustration of conventional SELEX. The high-affinity aptamers are amplified with PCR, while the low-affinity aptamers are removed along with the aptamers that interact with interfering substances, similar to the target. Created with BioRender (BioRender.com, 2025).

In the early 1990s, significant progress was made in the isolation of RNA motifs through the development of in vitro selection techniques. A landmark study by Tuerk and Gold demonstrated the first successful selection of RNA sequences against T4 DNA polymerase [17]. This pioneering approach gave rise to the method known as Systematic Evolution of Ligands by Exponential Enrichment (SELEX), an iterative procedure designed to enrich nucleic acid sequences with progressively higher affinity and specificity. A typical SELEX cycle consists of three fundamental steps: (1) incubating the target with a diverse sequence pool, (2) isolating the oligonucleotides that bind to the target, and (3) amplifying the bound sequences for subsequent rounds of selection. Modern oligonucleotide libraries used in SELEX contain up to ~10^15^ unique random sequences, enabling the identification of high-affinity ligands against virtually any molecular target [57,71,72]. The main steps of SELEX are also presented in Figure 4.

In structure-switching SELEX, an alternative to the conventional SELEX, candidate sequences are selected for their ability to undergo a target-induced conformational change that causes dissociation from a complementary capture strand and concomitant binding to the target [73]. FRELEX by NeoVentures Biotechnology Inc. incorporates the immobilization of thiolated aptamers on a gold surface. The target competes against similar-structured molecules for the aptamer selection. This last method holds the advantage of preserving the target’s interactions by immobilizing the aptamer instead, with a simple and less invasive procedure [74].

Finally, High-Throughput Sequencing-SELEX (HTS-SELEX) is an advanced technique combining the SELEX with Next-Generation Sequencing (NGS) technologies [75,76]. The main scope is to select and refine nucleotide aptamers, aiming for a higher binding affinity for a specific target molecule. HTS-SELEX requires several rounds of SELEX followed by Next-Generation Sequencing (NGS), allowing for the identification of thousands to millions of unique sequences that have been selected through the process [75]. Bioinformatics tools are used to analyze binding motifs, evolutionary trends, and sequence families, helping to pinpoint the most promising aptamers. Though SELEX methodology is widely used for choosing suitable aptamers, it comes undoubtedly with recognizable difficulties. First, SELEX is a time-consuming method, spanning weeks or even months of work until a successful candidate-aptamer is selected. Secondly, very few candidate aptamers are finally selected from the process, and even less could be finally synthesized for affinity characterization [77].

### 4.3. Aptamer Selection by Computational Methods and AI-Assisted Technology

The involvement of bioinformatics in aptamer selection and optimization has been introduced after the development of HTS-SELEX, thus presenting a so-called “in silico” approach in aptamer research [78]. In silico aptamer development follows two main stages: (1) candidate identification, wherein a subset of SELEX-derived sequences is selected on the basis of their strong interaction and binding potential with the target; (2) sequence optimization, in which those sequences that present an increased probability of strong interactions are evaluated employing structural modeling, docking, and molecular dynamics to effectively shorten, modify, and stabilize the selected aptamer candidates [78].

In silico aptamer selection is generally carried out through a four-step process: (1) Secondary structure prediction, in which the aptamer’s two-dimensional (2D) conformation is inferred from its nucleotide sequence. The accurate prediction of secondary structures—such as G-quadruplexes, hairpin loops, and T-junctions—is critical for estimating binding affinity to the target molecule and for informing subsequent tertiary structure modeling; (2) tertiary (3D) structure prediction, where the aptamer’s three-dimensional conformation is constructed and refined based on the secondary structure [79]; (3) molecular docking, which evaluates the binding behavior of small molecules within the binding sites of the target protein. Docking calculations, performed after tertiary structure modeling, provide detailed insights into the molecular interactions and binding affinities between the aptamer and its target [80]; and (4) molecular dynamics simulations, which assess both the stability and binding energetics of the aptamer–target complex by modeling atomic-level interactions over billions of time steps [79,81].

Besides the vast software options for computational analysis, the in silico aptamer selection comes with a lot of adversities. The most prominent challenge is that structure-based techniques need enormous processing power to evaluate the enormous volume of sequences produced by SELEX experiments. AI approaches can provide an appealing alternative for processing the massive information that is associated with aptamer selection and optimization. Predictive machine learning models—a type of AI technology—help in directed aptamer design by modeling the synthesis and evaluation of aptamer sequences in an in silico environment, thus drastically reducing the number of sequences that will eventually be screened in actual experiments [82]. It is not in the scope of the current presentation to fully present the tremendous capabilities of AI and machine learning, and for that, the reader is prompted to reviews dedicated to the subject [77,78,79].

### 4.4. Advantages of Aptamers Against Antibodies

Nucleotide aptamers and antibodies, both now employed in a plethora of experimental or practical applications aimed at diagnosis and therapy for AD, differ fundamentally in their structural composition. A summary of the key differences between aptamers and antibodies is included in Table 1. While antibodies are protein-based, consisting of amino acid chains, nucleotide aptamers are nucleic acid molecules. This structural divergence imparts distinct advantages to aptamers for specific applications. Despite the broad use of antibodies in diagnostics [83,84,85], aptamers represent a cutting-edge alternative. For instance, the T-SO508 DNA aptamer exhibits stronger and more specific binding to β-sheet structures of soluble amyloid oligomers compared to the A11 antibody [19]. However, aptamers have been shown to exhibit toxicity at low concentration levels and some degree of immunogenicity [57,86]. On the other hand, aptamers’ flexible size poses them as suitable medicinal carriers through the BBB, and more effectively than antibodies, allowing them to bind to smaller binding sites when encapsulated in exosomes or nanoliposomes coated with Rabies Virus Glycoprotein [86].

One crucial advantage of aptamers against antibodies is their rapid and cost-effective production. Aptamers are synthesized via a highly reproducible chemical process, ensuring both high purity and consistency without the use of animals, in full compliance with the 3Rs principle (Reduce, Replenishment, Refinement) [87,88,89]. In contrast, antibodies are typically produced in vivo, making their production more resource-intensive and time-consuming. Furthermore, aptamers are inherently more stable, demonstrating resistance to denaturation at elevated temperatures and maintaining functionality over extended periods, unlike antibodies, which are prone to losing their tertiary structure and activity under such conditions [57].

In diagnostic assays, aptamers offer significant advantages due to their ease of integration. Unlike antibody-based assays, which often involve complex immobilization procedures and multiple wash steps, aptamers can be employed in more straightforward, homogeneous formats. Additionally, aptamers can easily be conjugated to solid substrates—such as polymers, nanotubes, or nanoparticles—via electrostatic, hydrophobic, or covalent interactions, or by forming monolayers. Common linkers for aptamer attachment include biotin/streptavidin, avidin, neutravidin, as well as functional groups like amine, carboxyl, and thiol [90,91,92]. They can also be modified to carry detection molecules or therapeutic agents without disrupting their target interactions [93]. Notably, hybrid platforms combining AuNP–aptamer complexes with HRP-conjugated antibodies have achieved enhanced ELISA sensitivity for Aβ peptides by leveraging the color change induced by the secondary antibody, with detection ultimately mediated by the R18 aptamer [32,94].

**Table 1:** Comparison of the most significant properties of aptamers against antibodies.

Aptamers	Antibodies
DNA, RNA, or peptide molecules [93].	Proteins only [93].
High affinity, specificity, and thermal stability [93].	Their affinity is affected by denaturation due to pH and high temperature [93].
Low toxicity and immunogenicity [93].	Their presence causes immune response [93].
Ability to pass the BBB with their small size and easy modification [58].	Large molecules, affected by modifications [93].
Simple and automated synthesis through SELEX [93].	Expensive synthesis procedure that requires validation [93].
Their G-quadruplex structure detects the distinct hydrophobic areas of Aβ_40_ and Aβ_42_ [95].	Detect only the same hydrophilic surface of Aβ40 and Aβ42 and cannot recognize them [95].

## 5. Aptamers Targeting Alzheimer’s Disease Biomarkers

**Figure 5:**
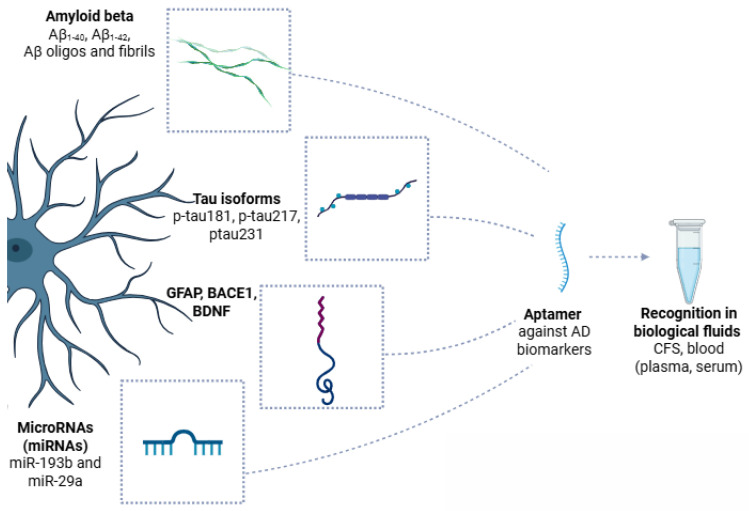
Schematic overview of Alzheimer’s disease (AD) biomarkers recognized by aptamers in biological fluids. Key pathological features of AD, including amyloid beta (Aβ) species (Aβ_40_, Aβ_42_, and Aβ oligomers), hyperphosphorylated tau isoforms (p-tau181, p-tau217, and p-tau231), glial fibrillary acidic protein (GFAP), beta-site APP cleaving enzyme 1 (BACE1), brain-derived neurotrophic factor (BDNF), and specific microRNAs (miR-193b and miR-29a), are secreted or released into cerebrospinal fluid, blood plasma, or serum. Aptamers, short single-stranded DNA or RNA molecules, are generated against these AD biomarkers, allowing for highly specific and sensitive recognition in biological samples. Created with BioRender (BioRender.com, 2025).

Following are the latest research data on the most key AD-related biomarkers, namely Aβ and tau species. A schematic overview of the recognition of AD biomarkers from aptamers and their employability in clinical analysis is also presented in Figure 5.

### 5.1. Aptamers Against Aβ

Aptamers have gained significant attention in Aβ biosensing and aggregation studies, particularly for their potential in detecting Aβ and inhibiting its aggregation. By targeting different forms of Aβ (monomers, oligomers, and fibrils), aptamers offer an alternative for early diagnosis and therapy. Currently, many nucleotide aptamers have been settled to target Aβ. A collection of the most employed aptamers against Aβ is depicted in Table 2. 

#### 5.1.1. RNA Aptamers

RNA aptamers are the first designed to target Aβ, providing affinities and specificities comparable to antibodies, with the added advantages of chemical modifications and reduced risk of contamination. The earliest RNA aptamer targeting Aβ, β55, was developed at early 2000, using as a target the Aβ_40_ monomer from a total of 18 RNA aptamers that were isolated with affinity column filtration. The β55 aptamer contains 107 nucleotides and has a Kd of 29 nM, selectively binding to the rich in β-sheet structures Aβ_40_ fibrils, rather than Aβ_40_ monomers [96].

In a later work, Takahashi et al. used Aβ_40_ bound to gold nanoparticles to model Aβ oligomers and thereby optimized the selection process. That approach yielded N2 and E2—two RNA aptamers that were later attached to gold nanoparticles. These nanoconjugates proved to bind both Aβ–AuNPs and free Aβ monomers (Kd values of 21.6 μM and 10.9 μM, respectively). The ability of N2 and E2 to bind Aβ monomers makes them promising candidates for detecting and modulating Aβ aggregation [97].

While early studies focused on Aβ monomers, later research revealed that Aβ oligomers are more cytotoxic than monomers or fibrils. This discovery shifted the focus to developing aptamers targeting Aβ oligomers. Rahimi et al. used Aβ_40_ oligomers as targets to develop RNA aptamers, specifically KM33 and KM41, via a nitrocellulose membrane filtration method. Adversely to what was expected, these syntheses presented no conjugation efficiency against Aβ_40_ oligomers, but rather to amyloid fibrils [98]. This outcome was further validated by repeating the selection using stabilized Aβ_40_ oligomers, with KM33 and KM41 aptamers emerging again as potent fibril binders [99]. Thus, these aptamers may prove promising for the selective pharmacological or imaging targeting of amyloid fibrils in vivo.

Given the central role of Aβ_42_ in AD, researchers have developed RNA aptamers that selectively recognize Aβ_42_ protofibrils. Using an Aβ_42_ protofibril model, E22P-AbD4, E22P-AbD31, and E22P-AbD43 were isolated; they bind protofibrils with Kd values on the order of 150 nM, with E22P-AbD43 presenting a strong preference for the Aβ_42_ dimer (Kd of ~20 nM), likely due to the G-quadruplex conformation that strongly promotes binding [100].

#### 5.1.2. DNA Aptamers

DNA aptamers are more cost-effective to synthesize and preserve, and generally more stable compared to RNA aptamers. These features are expected to enhance their specificity and functionality in complex environments, such as those required for in vivo applications and advanced analytical systems that involve challenging conditions (e.g., fluctuating temperatures, electric currents, magnetic fields, or mechanical stress). In 2012, eight DNA aptamers were found to be able to bind to Aβ_40_ oligomers. One aptamer, specifically T-SO508, exhibited a Kd of 25 nM for Aβ_40_ oligomers, which is lower than the respective Kd. It was later suggested that T-SO508 recognizes the β-sheet structures of Aβ oligomers, based on comparisons with the oligomer-specific antibody A11. T-SO508 is one of the most widely used aptamers as a capture/detection element across multiple platforms, enrichment and sensing assays, and as a probe to modulate in vitro aggregation kinetics [19].

Hoping to present a low-molecular-weight oligomer against Aβ_40_, Chakravarthy and colleagues developed the RNV95 DNA aptamer, which targets low-molecular-weight Aβ_40_ oligomers. This aptamer, identified through magnetic bead-assisted SELEX, forms a stable stem–loop structure comprising 39 bases. RNV95 can bind tetrameric and pentameric Aβ aggregates in hippocampal tissue, showing promising affinity-based assays for Aβ oligomer detection [26]. While both T-SO508 and RNV95 can specifically recognize Aβ oligomers, the selective binding of Aβ_40_ against Aβ_42_ oligomers is still considered as challenging when they are employed in aptamer-based sensing platforms for AD [19].

Three DNA aptamers specifically targeting the Aβ_42_ monomer were selected using magnetic bead-assisted in vitro screening. Among those, Aβ7-92-1H1, a 44-base aptamer, was optimized and shown to assume a stem–loop architecture with a strong specificity for the Aβ_42_ monomer (Kd = 63.4 nM), but not with the Aβ_40_ monomer. In addition, varying affinities for Aβ_42_ and Aβ_40_ aggregates, including oligomers and fibrils, have been demonstrated. Aβ7-92-1H1 selectivity for the Aβ_42_ monomer could be improved, but its ability to simultaneously recognize Aβ_42_ monomers and aggregates poses it as a promising candidate for developing inhibitors to regulate Aβ aggregation [19].

#### 5.1.3. Aptamers: A Tool to Detect Amyloid-Beta

Electrochemical sensors provide several advantages for Aβ detection, including low cost, straightforward miniaturization, and high sensitivity (often reaching the femtomolar range). Importantly, many electrochemical formats do not require sophisticated labeling strategies, although matrix-related interferences can limit performance in complex biological samples. Currently, various electrochemical platforms have been established to detect Aβ species, to study firstly their binding with their corresponding targets and secondly to monitor their aggregation in biological fluids [101,102]. These features have spurred extensive research into the electrical recognition of AD biomarkers [103,104].

Aptamers functionalized onto Au nanoparticles and copper organometallic structures that act as signal transducers were included in an aptasensing platform for detecting Aβ oligomers from the team of Zhou et al. This sensor demonstrated a broad analytical range from 1 nM to 2 µM and a low limit of detection (LOD) of 450 pM. The successful detection of Aβ oligomers in artificial CSF offered valuable insights into the potential for early diagnosis of AD [105]. In a subsequent study, Deng et al. developed an aptasensor specifically designed to capture Aβ40 oligomers via electrochemical signaling. This sensor was applied in both artificial CSF and human serum, demonstrating performance that met clinical requirements for Aβ40 oligomer detection [106].

The endeavor to detect AD pathological characteristics gave rise to another sensor, which employed a combination of antibody and aptamer technology, for the detection of Aβ_40_ and Aβ_42_ oligomers in artificial CSF, achieving an LOD of 100 pM [27]. The lowest LOD (0.27 pM) was brought by a biosensor combining molecular imprinted polymers and DNA aptamers designed to quantify Aβ_42_ oligomers [107].

Aiming for enhanced sensitivity, Liao et al. introduced a dual-amplification approach that combined exonuclease III-mediated DNA recycling with rolling circle amplification, enabling detection of Aβ oligomers at concentrations as low as 39 fM [108]. Furthermore, a complementary method exploiting the fluorescence quenching effect arising from resonance energy transfer between Ru(bipy)_3_^2+^ and gold nanorods was established, offering a detection window of 1.0 × 10^−5^ to 100 ng/mL and achieving a detection limit of 0.9 fM. This latter strategy has been successfully applied for the analysis of Aβ in CSF samples [109].

Another notable clinical application involved the use of a micron-scale organic electrochemical transistor integrated with a microfluidic platform, enabling the detection of Aβ aggregates across a broad dynamic range of 2.21 fM to 221 nM using just 1 µL of human blood serum [110]. This aptamer-based platform is distinguished by its low cost, design flexibility, operational simplicity, and wide applicability.

Fluorescence sensors are regarded as ultra-promising platforms in the quantitative analysis of Aβ species due to their high sensitivity and rapid response times. The combination of fluorescent molecules with aptamers provides an efficient detection tool that advances Aβ detection [111]. For instance, a fluorescence method utilizing DNA aptamers conjugated on Fe_3_O_4_ and upconverting nanoparticles (UCNPs) was developed for detecting Aβ oligomers. By combining magnetic separation with the luminescent response of UCNPs, a straightforward and highly sensitive method for Aβ detection was achieved, with a limit of detection as low as 36 pM. This method also proved applicable for detecting Aβ_40_ and Aβ_42_ oligomers in artificial CSF [112].

A variety of fluorescence, optical, and electrochemical aptasensors have been designed for Aβ detection with progressively improved sensitivity and applicability. For example, a fluorescence “off–on” assay employing FAM-labeled aptamers and molybdenum disulfide nanosheets (MoS_2_ NSs) quantified Aβ_42_ oligomers in hippocampal and cortical tissue of AD transgenic mice, with a linear range of 0.01–20 μM and an LOD of 3.1 nM. Interestingly, MoS_2_ NSs not only enhanced detection but also inhibited Aβ_42_ aggregation and promoted fibril degradation, highlighting their therapeutic potential [113]. Real-time in vivo imaging of Aβ oligomers was subsequently achieved with a three-dimensional DNA Walker nanoprobe, enabling discrimination between wild-type and AD mice with an LOD of 22.3 pM [114]. Similarly, a quadrivalent DNA nanostructure using cascaded catalytic hairpin assembly improved hybridization efficiency and stability, yielding an ultrasensitive LOD of 0.69 pM in real samples [115].

Fluorescence imaging also proved useful in histopathology: the β55 aptamer was shown by Farrar et al. to selectively stain Aβ plaques in AD mouse brains, co-localizing with thioflavin-S and revealing oligomeric structures surrounding dense plaque cores, supporting its role as a molecular imaging probe [61].

Beyond fluorescence-based methods, label-free optical strategies have gained traction due to lower cost and portability. Surface plasmon resonance (SPR) sensors, for instance, demonstrated high sensitivity and real-time analysis, with a dual aptamer system achieving detection limits of 0.2 pM for Aβ_40_ oligomers and 0.05 pM for fibrils in CSF [116,117]. Similarly, surface-enhanced Raman scattering (SERS) platforms enabled multiplex detection of Aβ_42_ oligomers and tau protein using aptamer–AuNP conjugates within 15 min [118]. Advanced fluorescence microscopy approaches, such as Total Internal Reflection Fluorescence Microscopy coupled with Electron Multiplying Charge-Coupled Device (TIRFM-EMCCD), further expanded multiplexing capacity, detecting Aβ_42_ monomers, tau 441, and p-tau181 at femtomolar levels while reducing assay costs [18].

Interference reflectance spectroscopy (IRS)-based biosensors exploit white light interference on thin nano- or microporous films. These sensors are advantageous due to their low cost, high sensitivity, and simple operational setup. For the first time, an aptamer sensor integrating nanoporous anodic aluminum oxide with IRS was developed to detect Aβ42 oligomers, covering a range of 0.5–50 μg/mL with a limit of detection of 0.02 μg/mL [119].

Colorimetric sensors have also gained attention because they are straightforward, inexpensive, and provide direct visual results. Zhu et al. designed a sensor for Aβ_40_ oligomers that relies on aptamer-induced aggregation of AuNPs under high-salt conditions, producing measurable changes in the absorption spectrum [120]. To enhance performance, a non-thiolated aptamer-based light-up sensor was constructed, enabling sensitive detection of low molecular weight Aβ_40_ oligomers even at 175 mM NaCl, making it suitable for point-of-care testing in complex biological environments [121].

ELISA, a common optical platform, detects signals through absorbance or fluorescence. Aptamers as probes paved the way for a novel ELISA approach that enables the detection of Aβ oligomers with greater binding affinity and capture efficiency compared to conventional antibodies. Incorporation of nanomaterials such as graphene oxide and gold nanoparticles further enhanced the assay, achieving a detection limit of 50 pM for Aβ oligomers. The dual-aptamer sandwich procedure improved performance compared to the antibody–aptamer sandwich by avoiding steric hindrance and epitope limitations [32].

Triple helix switches (THSs) combined with differential pulse voltammetry (DPV) provide another strategy for Aβ aptasensing. THSs utilize Hoogsteen base-pairing to introduce a third nucleotide strand into a duplex formed by two identical DNA sequences. By designing such structures to incorporate the target aptamer and AuNPs with a copper metal-organic framework DNA sequence (AuNPs@CuMOF/SD), strong DPV responses are generated. Upon binding of Aβ oligomers, the THS structure is disrupted, releasing AuNPs@CuMOF/SD and causing a reduction in DPV signals, with an LOD of 0.25 fM [122]. An alternative strategy utilizes a DNA aptamer immobilized on thionine-functionalized carbon nanomaterials, including reduced graphene oxide and multi-walled carbon nanotubes. Under optimized conditions, this aptasensor exhibited a concentration-dependent decrease in DPV current, achieving an LOD of 10 fM [123].

**Table 2:** Current referenced aptamers against Aβ: a detailed overview.

	Target	Aptamer	Sequence	Reference
RNA aptamers	Aβ_40_ fibrils	β55	5′-UUUACCGUAAGGCCUGUCUUCGUUUGACA-3′	[96]
	Aβ monomers	N2	5′-GGGAUGUUCUAGGCGGUUGAUGA-UAGCGUAUGCCACUCUCCUGGGACCCCCCGCCGGAUGGCCA-CAUCC-CAUCCAGAGUAGCAUAAUUGAUCCGA-3′	[97]
		E2	5′-GGGAUGUUCUAGGCGGUUGAUGA-UUUGGGGUGUCGGGCGAUUUUUAGGGUUGGGCCAGGCCGU-CAUCC-CAUCCAGAGUAGCAUAAUUGAUCCGA-3′	
	Aβ_40_ fibrils, Aβ_42_ fibrils, and other amyloid fibrils	KM33 and KM41	5′-TAATACGACTCACTATAGGGAATTCGA-GCTCGGTACC-3′	[98]
	Aβ_42_ protofibrils	E22P-AbD4	5′-GGGACGACCACCACCUGAUGGUCACGCCUUGGGGGAUCGACGUUUCCCACCUUGGCUGCC-3′	[100]
		E22P-AbD31	5′-GGGACGACCACCACC UGAUCGUACCACCGUUGCUAAUAA ACC UUU CUCCUUGGGGGAUCG-3′	
		E22P-AbD43	5′-GGGACGACCACCACC UGAUCGAGCUCACUUUCUACCUUUCCCACC UUCUUGGCUGCC-3′	
DNA aptamers	Aβ_40_ oligomers	T-SO508	5′-GCCTGTGGTGTTGGGGCGGGTGCG-3′	[124]
	Aβ_40_ oligomers	RNV95	5′-TGGGGGGCGGACGATAGGGGCCCCCCGGTAGGATGGACG-3′	[26]
	Aβ_42_ and Aβ_40_ aggregates	Aβ7-92-1H1	5′-CCGGTGGGGGACCAGTACAAAAGTGGGT AGGGCGGGTTGG AAAA-3′	[25]

#### 5.1.4. Aβ. Aptamer Applications in Therapeutics

Aptamers have demonstrated significant potential to slow AD progression by disrupting key stages of Aβ accumulation (see also Table 3). For instance, the β55 RNA aptamer selectively binds to β-sheet-rich amyloid fibrils of Aβ_40_, inhibiting their assembly into neurotoxic aggregates [96]. Subsequent studies should now confirm its application against oligomerization and fibrillization and subsequent mitigation of neurotoxicity in cellular and animal models. In addition, B55 has been conjugated with fluorescent tags, enabling imaging of amyloid deposits in human brain tissue and in vivo within APP/PS1 mouse models using two-photon microscopy [61]. Its ability to selectively recognize pathogenic Aβ conformers and modulate their aggregation highlights B55 as a promising therapeutic and diagnostic tool for AD.

Because aptamers can cross the blood–brain barrier, they can be decorated with therapeutic cargos. Resveratrol—a natural antioxidant—was incorporated into a cerium oxide nanoparticle platform coated with a phase-change material, ZIF-8, polydopamine, and an Aβ-targeting aptamer. This multifunctional construct both detects Aβ and delivers resveratrol to affected sites, combining diagnostic and therapeutic actions [125].

To improve binding to early Aβ protofibrils, researchers designed an aptamer from a mutant form of the Aβ_42_ dimer (where glutamate at position 22 was replaced by proline). This mutant inherently adopts a stable G-quadruplex conformation, giving rise to the aptamer E22P-AbD43, which exhibits strong binding to both Aβ_42_ monomers and dimers. In nucleation assays, it suppressed the appearance of oligomer peaks, showing that it disrupts early aggregation. Instead of forming protofibrils, the aggregation pathway was redirected toward spherical aggregates [86].

Carbon dots (CDs) functionalized with an Aβ-specific aptamer selectively accumulate at amyloid deposits. When exposed to red LED light, the CDs produce reactive oxygen species (ROS), which oxidize Aβ β-sheets, decrease peptide hydrophobicity, and inhibit further aggregation. This strategy provides precise spatiotemporal control, as ROS generation and Aβ denaturation occur only in the irradiated regions. In practice, it reduces plaque burden and promotes the formation of small, globular Aβ adducts instead of extended fibrils [126].

**Table 3:** Summary of the therapeutic applications and potential of Aβ-targeting aptamers.

Target	Aptamer	Type	Sequence	Reference
AChE	Ob2	DNA	5′-TAATACGACTCACTATAGCAATGGTACGGTACTTCCCTTCGAAAACACCCTGCCCCTCACACAAAAGTGCACGCTACTTTGCTAA-3’	[127]
Aβ oligomers	AβO Aptamer	DNA	GGTGGCTGGAGGGGGCGCGAACG	[125]
Aβ_42_ monomer and dimer	E22P-AbD43	RNA	5′-GGGACGACCACCACCUGAUCGAGCUCACUUUCUACCUUUCCCACCUUCUUGGCUGCC-3′	[86,100]
Aβ42 monomer	Aβ7-92-1H1	DNA	5′-CCGG TGGGGGACCAGTACAAAAGTGGGTAGGGCGGG TTGGAAAA-3′	[25]

### 5.2. Aptamers Against Tau Protein

Monitoring of tau protein isoforms, such as tau 441 (2N4R), tau 412 (1N4R), and tau 381 (1N3R), independent of phosphorylation status, has been widely studied [128]. Various aptasensor platforms, including colorimetric, interferometric, fluorescence, and electrochemical approaches, have been developed for the clinical detection of tau protein [20]. A detailed overview of the most employes aptamers against tau isoforms is presented in Table 4. 

Early efforts by Krylova et al. identified both dsDNA and ssDNA aptamers against tau 381 and tau 410, with the sequence 5′-GCGGAGCGTGGCAGG-3′ demonstrating the strongest affinity for tau 381 (low Kd values). Building on this, Lisi et al. introduced a rapid non-SELEX/capillary electrophoresis method, allowing the selection of aptamers against multiple tau isoforms (441, 381, 352, 383) in just one day. These aptamers were validated by SPR and fluorescence anisotropy, showing nanomolar detection limits (3–28 nM). This approach shortened the development timeline while producing aptamers with affinities comparable or superior to those obtained by traditional SELEX [131].

Almost a decade later, Kim et al. applied a tau 381 aptamer in an aptamer–antibody sandwich biosensor using SPR. The sensor achieved clinical relevance, detecting as little as 10 fM tau 381 in undiluted plasma—a 1000-fold sensitivity improvement compared to ELISA [132]. Other groups developed complementary approaches, successful but with lower sensitivity sensors employing the specific aptamer for tau 381 [130,133]. Shui et al. integrated an antibody–aptamer design with cysteamine-stabilized AuNPs and differential pulse voltammetry, reaching an LOD of 0.42 pM and validating its performance in sera from AD patients [133]. Tao et al. immobilized the aptamer on a graphene/thionine/AuNP-modified electrode, producing a DPV-based sensor with an LOD of 0.7 pM and the potential of differentiating AD patients from cognitively normal individuals [130].

Recent work has pushed clinical sensitivity even further. Hun and Kong engineered a photoelectrochemical biosensor by immobilizing the tau 381 aptamer on AuNP/MoSe_2_ nanosheets. Coupled with antibody detection and enzymatic amplification, this device achieved an ultra-low LOD of 0.3 fM, and, crucially, was effective in diluted human serum, successfully distinguishing AD patients from cognitively normal elderly subjects [134].

Ahn and Jang recently developed a liquid crystal-based aptasensor for detecting tau 381. The sensor operates via competitive binding between a tau-specific aptamer and poly-L-lysine (PLL). In the presence of tau, the aptamer preferentially binds the protein, freeing PLL to interact with 1.2-dioleoyl-sn-glycero-3-phospho-(1′-rac-glycerol) sodium salt, which induces a change in liquid crystal orientation. This alteration can be observed using polarized optical microscopy, allowing highly sensitive detection with LODs of 2.77 pg/mL in buffer, 10.86 pg/mL in serum, and 19.31 pg/mL in plasma [135].

Ziu et al. (2020) [136] designed a portable, real-time aptasensor for tau 441, employing biolayer interferometry in a “Dip-and-Read™” microwell format. Researchers have conjugated the Krylova tau aptamer with biotin–streptavidin chemistry on the sensor tips. The conjugation of the tau on the aptasensor results in interference in the white light reflection on the disposable fiber optic sensor of the module, which can then be analyzed. The sensor exhibited a LOD of 6.7 nM and demonstrated specificity toward tau sensing against Aβ_40_, α-synuclein, and bovine serum albumin (BSA) [136]. However, the authors do not report measurements under in vivo conditions (blood or other biological fluids) and do not discuss the limitation of the non-selectivity against tau 381, which the Krylova aptamer can also bind with similar potential [131].

Duan and colleagues have produced a sophisticated aptasensor for tau sensing based on a nanostructured polyvalent biotinylated aptamer scaffold (PBAS). First, researchers synthesized a linker DNA and then screened several biotinylated aptamers that could constitute the PBAS by partly hybridizing the linker. Magnetic beads carrying streptavidin were also employed, which can strongly interact with the PBAS due to biotin–streptavidin interaction and create a scaffold with numerous unhindered biotin sites. The successive addition of streptavidin–HRP that binds to these biotin targets leads to the production of a yellow catalytic color in the presence of the substrate tetra-methyl-benzidine. In the presence of tau, the protein binds to the aptamer, preventing PBAS–bead aggregation. This inhibition significantly reduces the enzymatic reaction, resulting in a weaker yellow signal or even a colorless outcome. The sensor demonstrated a low LOD of 153 pg/mL, high selectivity over BSA, casein, IgG, thrombin, and transferrin, and a short assay time of 50 min, with validated performance in artificial CSF [95].

### 5.3. Aptamers Against p-Tau

Given the growing importance of phosphorylated tau in AD diagnosis [137], several studies have focused on developing aptamers and aptasensors for its detection in biological fluids or for inhibiting its aggregation. Teng et al., for example, screened six aptamers targeting two phosphorylated epitopes of tau (Thr-231 and Ser-202) as well as full-length tau_441_. All selected aptamers exhibited strong binding affinities toward tau 441, with Kd values ranging from 5.5 to 68 nM [62]. Chan et al. reported a direct, ultrasensitive, multiplex antibody–aptamer sandwich immunoassay for the simultaneous measurement of AD-related bioanalytes, including p-tau181. The researchers have fabricated a magnetic nanoplatform based on silica-coated iron oxide nanoparticles, with conjugated antibodies against tau 441, p-tau181, and Aβ_42_. The sample to be analyzed is mixed with the nanoparticles alongside the aptamers, amplification probes, and the fluorophore (E)-1-(2-hydroxyethyl)-4-(2-(9-(2-(2-methoxyethoxyethyl)-9H-carbazol-3-yl)vinyl)pyridin-1-ium chloride (SPOH). Labeling of the magnetic hybrids by SPOH is then analyzed with a total internal reflection fluorescence microscopy electron-multiplying charge-coupled device. Analysis indicated that this system has significantly higher sensitivity than previous antibody-sandwich system (LODs: 8.4 fM (38 fg/mL), 4.3 fM (197 fg/mL) and 3.6 fM (165 fg/mL), for Aβ_42_, tau 441, and p-tau181, respectively), for quantification of the target biomarkers in both CSF and blood serum samples [18].

Phan and Cho have developed two alternative aptasensor systems for the measurement of p-tau231, with nitrogen-doped carbon dots (NCDs) or AuNPs. The study employed the aptamer developed for p-tau231 by Teng and colleagues [62], which was conjugated onto the surface of AuNPs or mixed with NCDs leading to significant fluorescence quenching. When p-tau231 is added, the peptide reacts with its corresponding aptamer and partially recovers NCD fluorescence intensity. Regarding the colorimetric sensor based on AuNPs, the binding of p-tau231 is detected by a copper-enhanced gold methodology inside microwells. In detail, the sample is added to the reaction chamber, followed by the addition of AuNP-aptamer nanoconjugates and a copper-enhancing solution. The reaction wells were then photographed and analyzed, and the normalized intensities were linear to the log[p-tau231]. The authors have found that the fluorescent NCD-based aptasensor presents an LOD of 3.64 ng/mL and the AuNP-based colorimetric assay an LOD of 4.71 pg/mL, and suggest that these sensors can be further explored as promising, fast tools for the detection of p-tau231 [30].

The same aptamer sequence was also employed in recent work of AuNP-coated glass carbon electrode sensors. The binding of p-tau231 on the sensor linearly changes the peak current with the log[p-tau231]. The detection of p-tau231 was specific, as no reaction was found for BSA, Aβ protein, CA19-9, γ-globulin, or fibrinogen. The sensor was stable, reproducible, and presented an LOD of 2.31 pg/mL. Furthermore, its performance was validated in human blood serum samples, achieving recovery rates between 97.59% and 103.26% [31].

### 5.4. Aptamers for Novel Biomarkers and Concluding Remarks

Novel aptamers have been identified against several well-validated AD biomarkers—like microRNA-193b (miR-193b), GFAP [138], brain-derived neurotrophic factor (BDNF) [139], BACE1 [140], and tumor necrosis factor-alpha (TNF-α) [141].Currently, Gli-233 and Gli-55 are the only aptamers reported in the literature for the specific targeting of GFAP protein and applied to astrocytomas. These DNA aptamers were originally selected from freshly resected astrocytomas of varying grades and subsequently characterized through computational and experimental approaches [138].

Chowdhury et al. identified eight DNA aptamers against BDNF through SELLEX. NV-B12 stood out for its sensitivity and selectivity for the binding target [139], but any clinical application has yet been reported exploiting this aptamer. Salehirozveh et al. developed the first electrical aptasensor to detect BDNF using an alternative DNA aptamer (5′-NH_2_-(T20)-GGATTTGAGCTTATGTGGCATAGGTTGCCTGGGTG GGTGGGGTCGGGGAA-3′). Owing to its high selectivity and specificity, the electrical aptasensor was highlighted by the authors as a promising tool for the early diagnosis and potential therapeutic monitoring of AD [142].

Wang and colleagues (2022) reported a BACE1-aptamer–modified tetrahedral framework nucleic acid (tFNA-Apt) and evaluated its therapeutic potential against AD using both in vitro assays and an APP-PS1 transgenic mouse model. The study showed that the tFNA scaffold acted as a brain-compatible carrier that enhanced delivery of the BACE1 aptamer to the central nervous system, leading to reduced production of amyloid-β peptides and attendant neurotoxic cascades; treated cells and animals also exhibited decreased ROS and reduced markers of apoptosis, consistent with neuroprotection. These results support the dual utility of tFNAs as both delivery vehicles and functional nanomedicines for nucleic-acid-based therapies and position the BACE1-aptamer–tFNA conjugate as a promising preclinical candidate for further translational development [140].

TNF-α has been widely targeted with DNA aptamers—most notably the 25 nt aptamer VR11—and integrated across multiple aptasensor architectures for sensitive cytokine quantification. Orava et al. first characterized VR11 (KD ≈ 7 nM) and showed it can block TNF-α signaling in vitro, establishing VR11 as a canonical TNF-α binder [143]. Electrochemical aptasensors functionalized with TNF-α aptamers have demonstrated direct detection in whole blood, with limits of detection on the order of 10^−11^–10^−12^ M (≈58 pM reported) and clinically relevant dynamic ranges [144]. More recent label-free solid-state devices—for example, asymmetric MoS_2_ diode sensors—have pushed sensitivity into the femtomolar range (≈10 fM) while retaining specificity in serum matrices [145]. Optical/kinetic methods such as biolayer interferometry have also been adapted into hybrid aptamer–antibody sensor platforms to enable real-time, highly specific quantification of TNF-α in serum. [146]. These primary studies and several recent reviews summarize the practical performance (LOD, linear range), immobilization chemistries, and matrix compatibility that make TNF-α aptamer-based sensors directly relevant to AD biomarker panels and point-of-care translation [146,147].

The discovery of miRNAs by Ambros and Horvitz in *C. elegans* [148,149] revealed how these small, 18–25-nucleotide, unstructured non-coding RNAs play a regulatory role in protein synthesis by influencing the stability, translation, or degradation of mRNA molecules [150]. Protected from degradation in bodily fluids by encapsulation in liposomes or exosomes [151], miRNAs are readily measurable in CSF and blood, thus making promising minimally invasive AD biomarkers. Therapeutic strategies now aim to restore miRNA levels using synthetic mimics or inhibitors. Two key miRNAs stand out: miR-29a downregulates BACE1 translation, and its downregulation correlates with increased Aβ burden [152]; and miR-193b directly targets the APP transcript, with reduced exosomal miR-193b detectable in prodromal AD [153]. To quantify these miRNAs in patient serum, researchers have developed highly sensitive, label-free nanosensors: gold-nanoparticle probes for miR-29a achieve femtomolar sensitivity without labels [154], while entropy-driven strand-displacement assays simultaneously read out changes in Aβ_42_ and miR-193b [155].

## 6. Conclusions

Looking ahead, combining the high selectivity of aptamers with emerging label-free technologies—such as SPR and electrochemical impedance spectroscopy—offers the potential to develop multiplexed panels for simultaneous detection of multiple AD biomarkers in minimally invasive samples. Integration with microfluidic platforms and wearable devices could further enhance point-of-care applications, enabling real-time monitoring of disease progression. Rigorous validation in large, longitudinal cohorts, together with correlation to neuroimaging and cognitive outcomes, will be critical to ensure clinical relevance and reproducibility. Beyond diagnostics, aptamer-based systems hold promise for therapeutic applications, including targeted delivery of drugs across the blood–brain barrier, gene-silencing constructs, or modulatory molecules, potentially allowing personalized interventions at early disease stages. By bridging biomarker detection with precision therapy, these platforms could pave the way for a fully integrated approach to AD management, from early diagnosis to intervention and monitoring of treatment efficacy.

## Figures and Tables

**Table 4:** Summarized overview of DNA aptamers against tau protein.

Target	Aptamer	Sequence	Reference
Tau protein isoforms (441, 381, 352, 383)	DNA aptamer 4618	5′-GCCTGTTGTGAGCCTCCTGTCGAACATGTTAATATTCCATCACCGACTTCTTTCTTGAGCGTTTATTCTTGTCTCCC-3′	[129]
	DNA aptamer 4133	5′-GCCTGTTGTGAGCCTCCTGTCGAACCTGTCAGGTCTTTGACGAGGCTTTTCTTCTTGAGCGTTTATTCTTGTCTCCC-3′	
	DNA aptamer 3914	5′-GCCTGTTGTGAGCCTCCTGTCGAACGGTTCTTAAGGCGTCCGTCTTCATTTGTTTTGAGCGTTTATTCTTGTCTCCC-3′	
	DNA aptamer 3146	5′-GCCTGTTGTGAGCCTCCTGTCGAACCTTTGGGGTGGCTTGACGAAGAAAGTAGTTGAGCGTTTATTCTTGTCTCCC-3′	
	DNA aptamer 433	5′-GCCTGTTGTGAGCCTCCTGTCGAAGGTGTCGACACCAGCCTTTAACCCTGTGTCTTGAGCGTTTATTCTTGTCTCCC-3′	
Tau 381	ssDNA-aptamer	5′-GCGGAGCGTGG CAGG-3′	[130]

## Data Availability

No new data were created or analyzed in this study.
